# Family visits in shared-housing arrangements for residents with dementia – a cross-sectional study on the impact on residents’ quality of life

**DOI:** 10.1186/s12877-015-0012-5

**Published:** 2015-02-25

**Authors:** Johannes Gräske, Saskia Meyer, Andreas Worch, Karin Wolf-Ostermann

**Affiliations:** Department 11: Human and Health Sciences, University of Bremen, PF 330440, 28334 Bremen, Germany

**Keywords:** Family involvement, Dementia care, Quality of life, Shared-housing arrangements

## Abstract

**Background:**

Shared-housing arrangements (SHA) are a German type of small-scale living arrangements for people with dementia (PwD). The involvement of family members is one core domain of SHA. But it has not been investigated yet, what are factors associated with family visits and if family involvement within SHA contributes to better residents’ quality of life (QoL).

**Method:**

A cross-sectional study including all SHA in Berlin/Germany was performed. Main parameters of interest were residents’ QoL (QUALIDEM) and frequencies of family visits within the SHA. Besides descriptive analyses we used logistic regression and ANCOVA to analyze the data.

**Results:**

58 SHA with 396 residents (78.4 years, 69.4% female) participated in the study. Older (OR: 1.034; 95% CI: 1.005; 1.064) and female residents (OR: 2.006; 95% CI: 1.018; 3.950) got more often visited by family members. An active participation of family members in SHA contributes on average to a better QoL in terms of social relationship and social isolation (all ANCOVA p < 0.005). A decreased QoL was found for people without family visits compared to those without family members.

**Conclusions:**

The involvement of family members in SHA is common but on a similar level compared to other care arrangements. Staff should convince available family members to visit PwD, in order to improve residents QoL. However, the response rate in the present study was about 13%, which may limit the results.

## Background

The number of people with dementia (PwD) is rising worldwide, with an estimated amount of 115 million PwD in 2050 [1]. Dementia is considered as one of the most expensive brain disorders [2] and frequently associated with admission to a nursing home [3]. However, the Organization for Economic Cooperation and Development (OECD) recommends, that care provision in a long-term care facility should be as homelike as possible [4]. A starting point for a concept including this guidance, was the group living in Sweden in the 1980s, which included the homelike aspect for the first time [5]. Today, similar concepts are to be found all over the world, e.g. Green Houses in the USA, group homes in Japan, small-scale living arrangements in the Netherlands, and German shared-housing arrangements (SHA) [6,7]. All these concepts are seen as a change from traditional care organized around nursing tasks to an individual approach of care. These arrangements are defined as “small and homelike and the care is person-centered with respect for residents’ needs and choice” [7]. Daily routines include meaningful activities to improve the principle of a normal living. Tasks focus on doing household chores, e.g. cooking, baking. Exemplarily, the German concept of SHA will be described in a more detailed way.

### Shared-housing arrangements

In Berlin/Germany the first SHA was founded in 1995 by family members of PwD [8], looking for alternative concepts of care and support. All care-dependent people with a care dependency package, regarding the German health-care insurance are eligible to live in such SHA’s. Today more than 1,400 SHA all over Germany exist, about 460 of them are situated in Berlin [6]. Typically, 6-8 residents - mostly with a diagnosis of dementia - share one large apartment. These apartments are located in ordinary apartment buildings, with non-care dependent tenants in the other apartments. A community health-care provider serves the residents of a SHA being part of a comprehensive network of further service providers, e.g. physiotherapists and general practitioners [9]. Residents of SHA are predominantly female care-dependent persons mostly with dementia and about 80 years old. Usually, they stay in the SHA until their end of life. Residents are not forced to move into a nursing home when the level of care dependency is increasing or they are about to die [9].

### Family involvement

Fischer et al. identified, that inclusion of family members is a major issue and one of the core domains in SHA [8]. This is in contrast to traditional nursing homes, were family caregiving is not explicit a major issue. Additionally, family members do not focus only on their own relatives, they also look after other residents in SHA. Family members are part of the group living in SHA and are supposed to act as legal representatives, if necessary. However, the emphasis of family inclusion into daily living is not a unique German issue, but inclusion addresses the wishes of family members in general [7]. The replacement of relatives into a care facility is an emotionally draining decision and triggers feelings of guilt and loss [10]. However, even after rehousing a family member in a care setting most relatives still want to be actively involved in care and want to visit people in need of care frequently [11]. The involvement of family members often establishes a relationship between the family and the health care team. Despite the shared goal of a sufficient quality of care for the resident, this relationship can be challenging. Sometimes registered nurses (RN) experience relatives as demanding [12]. Establishing an extensive information exchange and joint decision-making processes between formal and informal caregivers improves the involvement of family members in general [13-15]. Usually, family members serve as key persons for the affected people and the nursing staff. They are involved in all aspects of care provision, decision making, household tasks, trips outside the small-scale living arrangement and other meaningful activities [14,16-18]. Furthermore, family members are an important source in terms of biographical data, which are essential for a person-centered care [19].

Regarding international studies, the involvement of family members in the care of PwD is considered to be beneficial for PwD. Almost all PwD (81.0-87.0%) show once in a life a challenging behavior (e.g. apathy or aberrant behavior) [20]. The involvement of family members in dementia care reduces residents’ challenging behavior, and improves residents’ quality of life (QoL), as well as QoL of their family members [21-23]. In SHA typically meetings of family members among themselves as well as with the nursing staff are organized regularly to discuss issues affecting all aspects of group-living in SHA.

For family members, the concept of small-scale living facilities combines fewer burden and a higher satisfaction with the care situation compared to traditional nursing homes [24,25]. One way to quantify family involvement is to measure the frequency of visits and the tasks done by the family members [26]. In German SHA, 28.0% of the residents perceive a weekly visit by a family member, which is comparable to German special care units [27]. In homelike care arrangements in the Netherlands, the average numbers of visits are between 5.3 and 4.4 times per two week and on a comparable level as in traditional care arrangements [24]. In nursing homes in the US, most residents and family members come together at least once a week [28].

It is evident, that family care-giving in dementia care is a beneficial factor for PwD, even in care arrangements. However, for SHA only a few findings exist describing family involvement, including frequency and tasks done by family members. The impact on residents’ QoL, one of the major outcomes in dementia care, has not been investigated, yet. The present study aims to bridge this gap. Leading research questions are:

1. What is the frequency of family visits of PwD in SHA, and which tasks do family members perform?
2. What associations between presence or absence of family visits in SHA and residents’ characteristics can be found?
3. Is there an association of family visits on residents’ QoL in SHA?

## Methods

A cross-sectional study was performed, including all available SHA in Berlin, Germany.. Written standardized questionnaires were forwarded by post service to health care providers in SHA with the request to complete them. After four weeks, all providers got a kind reminder via phone. Usually, head nurses and social workers completed the questionnaire together and sent it back to the project team. Staff and residents received verbal and written information about the study, and verbal informed consent was obtained. This procedure is in line with German law, because personal data were collected and forwarded as anonymized data. Besides structural aspects of the SHA the questionnaire encompassed residents’ socio-demographics, their living circumstances (including care situation) before admission into the SHA, as well as the frequency of visits by family members. The tasks covered by family caregivers were evaluated with the minimum data set (MDS) [29]. However, only the tasks done within the whole group of residents were evaluated. With the respect to privacy, the task done only with their loved ones were not evaluated. And in addition, these tasks are mainly done in the private bedroom, where no nurse is present at this time. Therefore the validity would be at least questionable. The frequency of the visits was assessed in the following categories: no family members available, never or rarely, once to twice a month, several times a week, daily. Due to limitations in time and resources no detailed evaluation of the living situation of residents’ relatives was performed.

### Quality of life

QoL was assessed using the QUALIDEM [30,31] a proxy rated dementia-specific instrument which is appropriate to assess residents’ QoL in SHA [32,33], independently, whether they are with or without dementia. The QUALIDEM consists of 37 items assessing the frequency (‘never’ to ‘daily’) of certain residents’ behaviors. The instrument contains nine subdomains: *care relationship* (seven items), *positive affect* (six items), *negative affect* (three items), *restless tense behavior* (three items), *positive self-image* (three items), *social relations* (six items), *social isolation* (three items), *feeling at home* (four items), *having something to do* (two items). In accordance to Wetzels et al. for people with a severe level of dementia only 18 items and six subscales (*care relationship*, *positive affect*, *negative affect*, *restless tense behavior*, *social relations*, *social isolation*) were applied [34]. In addition, to the subscales an *overall QoL* score was calculated by summing all applicable items. The sum scores of the subdomains and the *overall QoL* were linear adapted to a scale with a range from 0-100. Higher scores indicate a better QoL.

### Challenging behavior

Challenging behavior was assessed by using the Cohen-Mansfield Agitation Inventory (CMAI) [35]. Nursing staff rated 29 behaviors on a 7-point Likert scale (from ‘never’ to ‘a few times in an hour’). The analysis indicated whether aggressive behavior, physically non-aggressive behavior or verbally agitated behavior occurred [36]. Additionally, it was calculated whether a resident showed at least one challenging behavior, either aggressive behavior, physically non-aggressive behavior or verbally agitated behavior or even a combination of these behaviors.

### Severity of dementia

The level of dementia was assessed using the Global Deterioration Scale (GDS) [37]. The range is from one (no cognitive decline) to seven (very severe cognitive decline). Because of the fact, there are just few residents with a GDS lower than six, we summarized the categories of five and below to one category.

### Data analysis

The data were analyzed descriptively, screening also for missing values and outliers using SPSS® 22. For metric and/or ordinal variables, Pearson and Spearman correlations were performed. Chi-square tests, Fisher’s exact test, Mann-Whitney U tests, t-tests and Kruskal Wallis test were used to analyze the data.

A logistic regression was performed to identify associations for receiving visits of family members. The independent variables were: sex [mal/female], age [in years], at least one challenging behavior [CMAI: no/yes], medical diagnosis of dementia [no/yes], severity of dementia [GDS: ≤ 5, 6 7], and living circumstances before the admission to the SHA [alone/with family member or partner]. The logistic regression did not include interaction terms between.

The impact of visits of family members is analyzed using ANCOVA models to explain the QoL including all subdomains of the QUALIDEM and the *overall* score. Independent variables were residents’ sex [male/female], age [in years], severity of dementia [GDS: ≤ 5, 6 7], and occurrence of at least one challenging behavior [CMAI: no/yes]. Additionally, frequencies of visits by family members [daily, several times a week, monthly, never] were taken into account. Because of the small number of participants, interactions between independent variables were not modeled. Before conducting further analyses, statistical model assumptions were examined.

Statistical significance is specified as p ≤ 0.05. Because of multiple testing Bonferroni-correction was applied for QUALIDEM analyses (overall score and subscales; p ≤ 0.005).

### Ethical considerations

The ethics committee of the German Society of Nursing Sciences approved the study protocol.

## Results

58 SHA and 396 residents participated in the study which corresponds to a response rate of 12.8% of all existing SHA in Berlin and 15.2% of all estimated 2,600 residents in SHA in Berlin (source population). The main reasons mentioned for not taking part in the study were lack of time and interest. The mean number of residents per SHA was 6.9 (SD 2.2) with a range from three to twelve.

### Sample characteristics

Residents’ socio-demographic characteristics, challenging behavior and QoL scores of are shown in Table 1. There was a strong significant correlation between age and length of stay in the SHA (Pearson’s r 0.981, p < 0.001). Women (81.0 years) were significantly older than men (72.5 years) (*t*-test, p < 0.001). Women were more often with dementia (Fisher’s exact test, p < 0.001) and show a more severe level of dementia relating to the GDS (Chi square test, p = 0.009). 57.8% (n = 229) of all residents were with at least one challenging behavior. Residents with a mild to moderate severity of dementia (71.1 years) were significantly younger than residents with a severe (79.2 years) and very severe level of dementia (81.0 years) (ANOVA, p < 0.001). The length of stay was shorter (2.3 years) for residents with a severe level of dementia (GDS 6) than for those with a mild to moderate (2.8 years) and very severe level (3.1 years) (ANOVA, p = 0.013). Residents with dementia significantly more often exhibited at least one challenging behavior (Fisher’s exact test, p < 0.001). The severity of dementia was not related to challenging behavior (Chi square test, p > 0.460).

**Table 1:** **Characteristics of study sample**

	**Residents (n = 396)**
**Age** in years; mean (SD)	78.4 (11.1)
**Women** in % (n)	69.4 (275)
**Time being in the SHA** in years; mean (SD)	2.7 (2.3)
**Medical diagnosis of dementia** in % (n)	71.0 (281)
**Severity of dementia** (GDS) in % (n)	
≤ 5 (moderately severe cognitive decline)	15.9 (63)
6 (severe cognitive decline)	42.2 (167)
7 (very severe cognitive decline)	38.4 (152)
**Challenging behavior** (CMAI) in % (n)	
Physically nonaggressive behavior	34.3 (136)
Verbally agitated behavior	37.9 (150)
Aggressive behavior	15.9 (63)
At least one need-driven behavior	57.8 (229)
**With family members** in % (n)	65.4 (259)
**Weekly family visits** in % (n)*	44.8 (116)
**Living situation before admission into SHA** in % (n)	
Alone	65.7 (260)
With family member/partner	27.0 (107)

*of residents with family members; GDS: Global Deterioration Scale, CMAI: Cohen-Mansfield Agitation Inventory; SHA: Shared-housing arrangement.

Before admission to a SHA, most of the residents (65.7%, n = 260) were living independently without sharing their apartment with any family member or partner. These residents were significantly older (79.6 years) than other residents (74.8) (*t*-test, p < 0.001). There were no differences concerning sex, the presence of a medical diagnosed dementia syndrome and the living situation (all Fisher’s exact test, p > 0.05).

### Quality of life

Generally, QoL was on a moderate level (see Table 2) and for most domains including the total QoL score, no differences for sex could be established (all *t*-test, p > 0.05). Age is partly associated with residents’ QoL. A week positive correlation was found for *care relationship*, *restless tense behavior* (all Pearson r, p < 0.05) and a weak negative correlation for *having something to do* (Pearson r -0.210, p = 0.012). People with lower severity of dementia showed better *overall* QoL, *negative affect, restless tense behavior, positive self-image, social isolation* and *having something to do* (all ANOVA, p < 0.05).

**Table 2:** **Resident’s Quality of Life (QUALIDEM)**

	**mean (SD)**
**Overall QoL**	69.5 (14.1)
**Care relationship**	72.1 (21.0)
**Positive affect**	73.1 (23.6)
**Negative affect**	71.6 (23.7)
**Restless tense behavior**	63.2 (30.1)
Positive self-image*	75.5 (23.1)
**Social relationship**	68.8 (21.9)
**Social isolation**	69.4 (22.4)
Feeling at home*	80.7 (18.2)
Having something to do*	56.2 (26.5)

*not applied for Global Deterioration Scale 7; for all scales 0-100, higher scores indicate better quality of life; QoL: quality of life.

### Family caregiving

**Figure 1:**
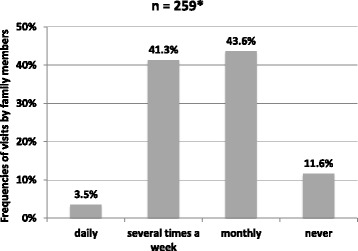
**Number of family visits.**

For 65.4% of all 396 residents, family members could be identified. Of those 11.6% (n = 30) did not get family visits, at all. Frequencies of visits of family members were not related to residents’ sex, medical diagnosis of dementia, the living situation admission into the SHA (all Mann-Whitney-U, p > 0.05), and the severity of dementia (Kruskal Wallis, p = 0.127). Group differences were found concerning age. Persons, who receive daily visits by their family members, were significantly older, than with less visits (ANOVA, p < 0.001) Figure 1.

44.8% (n = 116) of the residents with identified family members (29.3% of all residents) received family visits at least once a week. Tasks done by family members are displayed in Table 3. On average, family members do 3.7 (SD 2.3) different tasks per week, e.g. talking in the group or going for walk.

**Table 3:** **Tasks (MDS) done by family members**

**Task**	Proportion of involved family members (n = 259)*; % (n)
Talking in the group	76.8 (199)
Walks	53.3 (138)
Go on errands**	48.3 (125)
Room decoration**	37.8 (96)
Family members meeting**	33.2 (86)
Trips	33.2 (86)
Reading	28.6 (74)
Nursing tasks**	23.9 (62)
Music	21.6 (56)
Cooking, baking, doing household chores	11.6 (30)

*only for residents with family members, **tasks in addition to the Minimum Data Set.

### Factors associated with weekly family visits

A logistic regression was performed to estimate factors associated with weekly family visits. Including all assumed resident-related influencing variables (age, sex, medical diagnosis of dementia, severity of dementia, at least one challenging behavior) into the model of logistic regression, 59.3% of all cases could be classified correctly. However, only 12.0% of the estimated variance could be explained (Nagelkerkes R^2^ = 0.120). Significant factors were age (OR: 1.034; 95% CI: 1.005; 1.064, p = 0.021) and being female (OR: 2.006; 95% CI: 1.018; 3.950, p = 0.044). This means, the odds to receive weekly family visits rise with an increased age or being female. All other assumed variables, including whether the residents lived together with a family member or partner before moving into the SHA, were not explaining weekly family visits (all, p > 0.05).

### Impact of family involvement

The frequency of visits of family members did not correlate with most QUALIDEM scores (all Spearman-Rho, p > 0.05). Only for the subdomain *social isolation* a weak positive correlation was found (Spearman-Rho 0.156, p = 0.013). This is the only subdomain, where significant group differences between the frequencies of visits were found. Residents with family members who are never visited by the family members had a significantly lower QoL (55.9) in this subdomain (ANOVA, p = 0.005), which was even lower than for residents without family members (69.1). The group with the highest scores in this subdomain were residents with several visits per week (72.1).

The multivariate analyses (adjustments for challenging behavior, severity of dementia, sex and age) showed similar results. For the *overall QoL* (β -5.054) and *social isolation* (β -14.720) residents, who have family members never visited them, showed a significant lower QoL than residents without family members do. For the subdomain *social relationship*, residents with monthly visits (β 8.494) showed a higher QoL compared with residents without family members (see Tables 4 and 5).

**Table 4:** **Factors associated with QoL (QUALIDEM)**

**Dependent Variable**	**p-value (Model)**	**R** ^**2**^	**Significant influencing variable**	**p-value, partial Eta** ^**2**^ **-coefficient**
QUALIDEM **overall QoL** non-significant independent variable: 3 (see underline)	< 0.001	0.208	At least one challenging behavior (CMAI)	< 0.001, 0.136
			Severity of dementia (GDS)	0.018, 0.056
			Family visits	0.022, 0.031
			Age	0.004, 0.023
QUALIDEM **care relationship** non-significant independent variable: 2, 3, 4, (see underline)	< 0.001	0.159	At least one challenging behavior (CMAI)	0.001, 0.115
			Age	0.003, 0.023
QUALIDEM **positive affect** non-significant independent variable: 1, 3, 4, 5 (see underline)	0.034	0.048	Severity of dementia (GDS)	0.020, 0.034
QUALIDEM **negative affect** non-significant independent variable: 4, 5 (see underline)	0.001	0.145	At least one challenging behavior (CMAI)	0.001, 0.075
			Severity of dementia (GDS)	0.013, 0.023
			Sex	0.003, 0.024
QUALIDEM **restless tense behaviour** non-significant independent variable: 3, 4 (see underline)	< 0.001	0.188	At least one challenging behavior (CMAI)	0.001, 0.068
			Severity of dementia (GDS)	0.001, 0.113
			Age	< 0.001, 0.033
QUALIDEM positive self-image* non-significant independent variable: 4 (see underline)	< 0.001	0.122	At least one challenging behavior (CMAI)	0.004, 0.037
			Severity of dementia (GDS)	0.011, 0.030
			Sex	0.033, 0.021
			Age	0.034, 0.021
QUALIDEM **social relationship** non-significant independent variable: 3, 4 (see underline)	< 0.001	0.089	Sex	< 0.001, 0.041
			Family visits	0.050, 0.026
QUALIDEM **social isolation** non-significant independent variable: 3, 5 (see underline)	< 0.001	0.157	At least one challenging behavior (CMAI)	< 0.001, 0.132
			Severity of dementia (GDS)	0.040, 0.029
			Family visits	0.002, 0.045
QUALIDEM feeling at home* non-significant independent variable: 1, 2, 3, 4, 5 (see underline)	0.088	0.088	n/a	n/a
QUALIDEM having something to do* non-significant independent variable: 1, 2, 3, 4, 5, (see underline)	0.010	0.087	n/a	n/a

1: challenging behaviour (CMAI), 2: severity of Dementia (GDS), 3: residents’ sex, 4: family visits, 5: residents’ age, *not for people with very severe dementia (GDS 7), n/a not applicable; CMAI: Cohen-Mansfield Agitation Inventory; GDS: Global Deterioration Scale.

**Table 5:** **Regression coefficients of significant associated factors of QUALIDEM-ratings**

**Independent variables**		**Dependent variables (β; p; [95% CI])**				
		**QUALIDEM overall QoL**	**QUALIDEM care relation-ship**	**QUALIDEM positive affect**	**QUALIDEM negative affect**	**QUALIDEM restless tense behavior**
Resident’s sex	Male*	n.s.	n.s.	n.s.	-7.974; 0.003 [-13.215; -2.732]	n.s.
Severity of dementia (GDS)	GDS ≤ 5**	9.042; <0.001 [5.075; 13.009]	n.s.	7.906; 0.033 [0.669; 15.154]	8.333; 0.018 [1.437; 15.230]	23.562; <0.001 [15.001; 32.122]
	GDS = 6**	4.182; 0.004 [1.341; 7.024]	n.s.	6.594; 0.013 [1.402; 11.786]	-1.761; 0.484 [-6.700; 3.179]	18.525; <0.001 [12.394; 24.657]
Minimum 1 challenging behavior (CMAI)	No***	10.212; <0.001 [7.564; 12.860]	14.401; <0.001 [18.622; 51.759]	n.s.	12.765; <0.001 [8.161; 17.369]	15.036; <0.001 [9.322; 20.751]
Frequencies of family visits	Family visits: daily****	-1.947; 0.658 [-10.596; 6.702]	n.s.	n.s.	n.s.	n.s.
	Family visits: several times a week****	1.814; 0.288 [-1.541; 5.168]	n.s.	n.s.	n.s.	n.s.
	Family visits: monthly****	3.216; 0.255 [-0.074; 6.506]	n.s.	n.s.	n.s.	n.s.
	Family visits: never****	-5.054; 0.050 [-10.111; 0.003]	n.s.	n.s.	n.s.	n.s.
Residents age^§^		0.196; 0.004 [-0.064; 0.329]	0.307; 0.003 [0.102; 0.512]	n.s.	n.s.	0.510; <0.001 [0.225; 0.796]
**Independent variables**		**Dependent variables (β; p; [95% CI])**				
		**QUALIDEM positive self-image** ^**#**^	**QUALIDEM social relations**	**QUALIDEM social isolation**	**QUALIDEM feeling at home** ^**#**^	**QUALIDEM having something to do** ^**#**^
Resident’s sex	Male*	-7.241; 0.033 [-13.896; -0.587]	10.086; <0.001 [5.059; 15.114]	n.s.	n.s.	n.s.
Severity of dementia (GDS)	GDS ≤ 5**	8.922; 0.011 [2.095; 15.748]	n.s.	10.368; 0.002 [3.929; 16.807]	n.s.	n.s.
	GDS = 6**	(reference)	n.s.	5.020; 0.033 [0.408; 9.632]	n.s.	n.s.
Minimum 1 challenging behavior (CMAI)	No***	8.657; 0.004 [2.769; 14.546]	n.s.	16.293, <0.001 [11.995; 20.591]	n.s.	n.s.
Frequencies of family visits	Family visits: daily****	n.s.	1.948; 0.791 [-12.474; 16.371]	6.133; 0.391 (-7.905; 20.172 0.391	n.s.	n.s.
	Family visits: several times a week****	n.s.	2.423; 0.395 [-3.171; 8.016]	0.753 (-4.691; 6.198) 0.786	n.s.	n.s.
	Family visits: monthly****	n.s.	8.494; 0.002 [3.008; 13.981]	2.227; 0.413 [-3.113; 7.568]	n.s.	n.s.
	Family visits: never****	n.s.	2.066; 0.482 [-6.367; 10.498]	-14.720; <0.001 [-22.929; -6.512]	n.s.	n.s.
Residents age^§^	0.311 (0.024; 0.598) 0.034	n.s.	n.s.	n.s.	n.s.	

*as compared to female **as compared to most severe dementia (GDS 7); ***as compared to at least 1 need-driven behavior; ****as compared to no family members available; ^§^ continuous co-variable; ^#^not for people with GDS 7; GDS: Global Deterioration Scale; CMAI: Cohen-Mansfield Agitation Inventory; n.s.: not significant.

## Discussion

Due to the fact, that dementia care will become a challenge in the next years it is important to identify approaches, which contribute to a better QoL of PwD. The aim of the present paper was to analyze the effect of family involvement in SHA on resident’s QoL. This and the comparability of the characteristics of the study sample to those of prior studies indicate that typical residents of SHA are included into the study [9]. Therefore, it is to assume that there is no sample bias.

### Frequencies of family visits

The family involvement in the included sample is more extensive than in prior studies. In German SHA it is described, that 28.0% of the residents perceive a weekly visit by their family members [27]. In the present study, the proportion is higher (44.8%), but comparable to findings from similar care arrangements in the Netherlands [24]. Even findings from traditional nursing homes are comparable [28]. This means, that although family involvement is one core domain of SHA, it is not more intensive than in traditional nursing homes. However, a final conclusion, whether there might be differences of family caregiving between SHA and traditional nursing homes can not be drawn based on the present results. The investigation of different aspects, such a direct comparison between both settings, still is missing.

### Factors associated with weekly family visits

Female residents more often get weekly visits (OR: 2.006, compared to men). One explanation could be that many women are widowed and receive visits and help from their adult children especially their daughters which are usually the primary family caregivers. Additionally, the bound between mothers and daughters is mostly stronger than in any other relation [38,39]. It is surprising, that whether the residents lived together with family members or partners before admission to the SHA or not is not associated the frequency of visits of family members. It is known, that family members wish to stay involved into the care of their loved ones after the admission to a care arrangement [40]. Therefore it could be expected, that significantly more people, living together with a family member/partner prior to moving into the SHA, would have been visited. However, the conducted analyses, to identify associations of weekly family visits should be interpreted with prudence. Only characteristics of the residents were taken into account. No detailed information was available concerning characteristics of family members, whether they lived in Berlin or were even able to visit the resident. There could be reasonable circumstances, why family visits are precluded, e.g. being bound to bed. An implication for future studies is to consider this information as being worth to be included into the study design.

### Impact of family involvement

Residents without identified family members in our study still had a higher QoL at least in the subdomain *social isolation* and *overall QoL* than residents with family members but without visits of them. It is remarkable although both scores included further aspects than only family visits. Referring to this, the nursing staff should get in contact with those and improve their communication to the family members. There might be reasons, why they are not visiting the residents, e.g. being bound to bed, not living in the area or even familiar conflicts.

A large proportion of residents does not have close relatives anymore, although one core domain of SHA is the involvement of family members. In the present study, it is unknown, whether these residents were without family members, when moving into the SHA or not. In any case, to ban a person from moving into a SHA because of family background would not be acceptable for ethical reasons.

To improve the family involvement into the care in SHA, the “Partners in Caregiving” could be a promising approach. The program is based on an improved communication between family members and professional caregivers and on conflict resolution skills [41]. Concerning residents who do not receive family visits for any reason, there is the option to involve volunteers. The collaboration with e.g. the Alzheimer’s Disease Associations could be a promising approach to recruit people who already are in contact with people with a progressive cognitive decline.

## Conclusion

The present results show, that the involvement of family members in the routine of SHA, is associated with better residents’ QoL. This is in line with prior knowledge. However, the present study adds new knowledge in family nursing. No visits of family members are associated with lower QoL of PwD compared to those, who do not have family members anymore. Therefore, health care providers should support family visits and encourage relatives and friends to visit residents. Nevertheless, this broad field of family participation in dementia care has to be investigated more intensively, e.g. in terms of a direct comparison with other care settings or the inclusion of further factors related to family characteristics.

### Limitations

It was demonstrated that family involvement is less present in SHA as postulated, but is associated in general with better residents’ outcomes. Nevertheless, some limitations have to be stated, before generalizing these findings. First, a relatively large sample size was included into the study, but this sample was regionally limited to Berlin and only represents about 15% of the source population. Second, there is still a large discussion about the level of agreement between self- and proxy ratings in QoL measurements [42,43]. Furthermore, we did not examine characteristics of family members (e.g. living situation, distance to the SHA) and their time spend within the SHA. This might influence the results stated in this publication.

## Abbreviations

AN(C)OVA: Analysis of (co-)variance
CMAI: Cohen-Mansfield Agitation Inventory
GDS: Global Deterioration Scale
MDS: Minimum Data Set
OR: Odds Ratio
PwD: Person with dementia
QoL: Quality of life
RN: Registered nurse
SD: Standard deviation
SHA: Shared-housing arrangement
